# Granulocytic sarcoma of the pancreas on ^18^F-FDG PET/CT: A case report

**DOI:** 10.1097/MD.0000000000005570

**Published:** 2016-12-09

**Authors:** Akira Ishii, Tadakazu Kondo, Tomomi Oka, Yuji Nakamoto, Akifumi Takaori-Kondo

**Affiliations:** a Departments of Hematology and Oncology; b Diagnostic Imaging and Nuclear Medicine, Graduate School of Medicine, Kyoto University, Kyoto, Japan.

**Keywords:** ^18^F-FDG PET/CT, granulocytic sarcoma, pancreas

## Abstract

**Rationale:**

Granulocytic sarcoma (GS) is defined as leukemia infiltration in any organ other than the bone marrow. GS rarely occurs in the pancreas. Here, we present the first report of GS in the pancreas on ^18^F-fluorodexyglucose positron emission tomography/computed tomography (^18^F-FDG PET/CT).

**Patient concerns:**

A 19-year-old male patient with acute myeloid leukemia received a human leukocyte antigen-haploidentical stem cell transplant as a second transplant while in second complete remission.

**Interventions:**

After a second stem cell transplant, obstructive pancreatitis accompanied by a mass in the pancreatic head was observed. FDG-PET/CT revealed abnormal activity in the head of the pancreas and the skin in the patient's left breast area.

**Diagnoses:**

Pathological examination demonstrated relapsed acute myeloid leukemia in both the lesions.

**Outcomes:**

This is the first report showing the ^18^F-FDG PET/CT findings of GS in the pancreas.

**Lessons:**

^18^F-FDG PET/CT may help determine the stage of GS.

## 1 Introduction

Granulocytic sarcoma (GS) is defined as leukemia infiltration in any organ other than the bone marrow. GS of the pancreas is rare presentation of acute myeloid leukemia relapse. Sometimes, it occurs after allogeneic transplantation, presumably reflecting weaknesses of graft-versus-leukemia effect in the extramedullary tissues. Here, we present the first report of GS in the pancreas on ^18^F-fluorodexyglucose positron emission tomography/computed tomography (^18^F-FDG PET/CT).

## 2 Case report

A 19-year-old male patient with acute myeloid leukemia received a human leukocyte antigen-haploidentical stem cell transplant as a second transplant while in second complete remission. The conditioning regimen consisted of total body irradiation, melphalan, fludarabine, and cytarabine.

**Figure 1:**
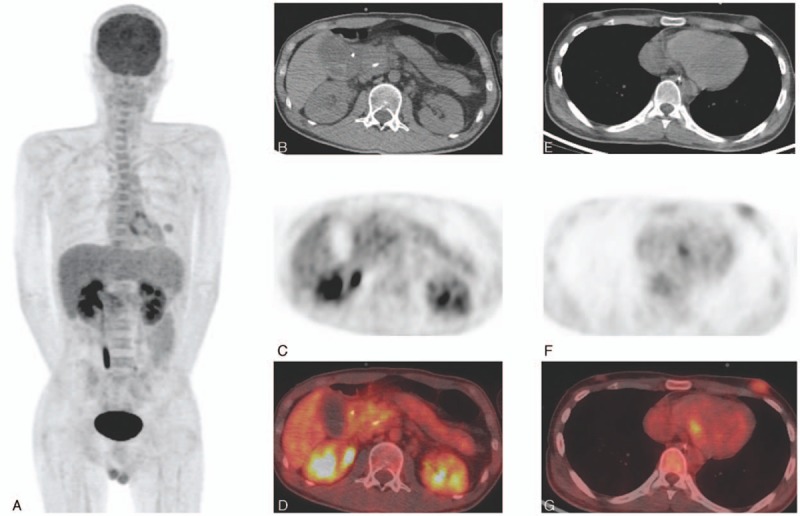
Imaging findings on ^18^F-fluorodexyglucose positron emission tomography/computed tomography. Three focal lesions (A) with moderate metabolic activity were observed in the pancreatic head ([B], computed tomography [CT]; [C], positron emission tomography [PET]; [D], fusion), in the skin in his left breast area ([E], CT; [F], PET; [G], fusion), and in the abdominal skin.

Five months after the transplant, he developed epigastric and back pain, and a skin tumor appeared on the abdomen. Laboratory analyses showed elevated amylase (935 U/L) and lipase (2547 U/L) levels. CT revealed distension of the Wirsung duct and swelling of the pancreas. Diffusion-weighted magnetic resonance imaging demonstrated restricted diffusion in a portion of the pancreatic head, corresponding to a pancreatic mass. Abdominal skin biopsy and endoscopic ultrasound-guided fine needle aspiration of the pancreatic mass revealed tumor cells positive for the myeloid marker myeloperoxidase and CD34, as determined by immunohistological examination. Furthermore, ^18^F-FDG PET/CT was also performed. Although the blood glucose level was not measured before the examination, in spite of his steroid diabetes, 3 focal lesions (Fig. 1A) with moderate metabolic activity were observed in the pancreatic head (standardized uptake value maximum [SUV max], 3.5) (Fig. 1B, CT; C, PET; D, fusion), in the skin in his left breast area (SUV max, 2.6) (Fig. 1E, CT; F, PET; G, fusion), and in the abdominal skin (SUV max, 1.5). A diagnosis of relapsed acute myeloid leukemia involving the pancreas and the skin was made, and salvage chemotherapy was consequently initiated.

## 3 Discussion

This is the first report showing the ^18^F-FDG PET/CT findings of a GS in the pancreas. The differential diagnosis between pancreatic GS and other diseases using ^18^F-FDG PET/CT is limited when the tumor is accompanied by obstructive pancreatitis. GS is defined as leukemia infiltration in any organ other than the bone marrow. GS develops in 9% of patients with acute myeloid leukemia^[1]^ and occurs in 5% to 7% of patients undergoing allogeneic hematopoietic stem cell transplantation as a relapse.^[2]^ Only approximately 20 cases of pancreatic GS have been reported.^[3–15]^ In almost all cases, CT and/or magnetic resonance imaging were performed, whereas the ^18^F-FDG PET/CT findings remain unknown. The reported ^18^F-FDG PET uptake of GS ranges between SUVmax 2.6 and 9.7,^[16]^ which may overlap with that in other pancreatic cancers and inflammatory responses.^[17]^ Because of these overlaps, it may be difficult to distinguish GS from other diseases if uptake is detected in the pancreas. In our case, obstructive pancreatitis accompanied GS, possibly making it more difficult to distinguish malignancy from an inflammatory response. Moreover, the uptake in the pancreas might have been decreased by steroid diabetes in our case. Thus, while ^18^F-FDG PET/CT may be useful to detect GS in the early stage, determine the stage, and evaluate the treatment response,^[18]^ if GS develops in the pancreas, especially when accompanied by pancreatitis, ^18^F-FDG PET/CT shows limited usefulness for the diagnosis. Accordingly, it is necessary to perform biopsy to confirm the diagnosis, similar to that performed in our case.

In conclusion, this is the first report showing the ^18^F-FDG PET/CT findings of GS in the pancreas; ^18^F-FDG PET/CT may help determine the stage of GS.

## 4 Consent

Informed consent was signed by the patient for the publication of this report and related images.
